# Low-Dose Tolvaptan for the Treatment of Dilutional Hyponatremia in Cirrhosis: A Case Report and Literature Review

**DOI:** 10.1155/2014/795261

**Published:** 2014-04-30

**Authors:** Guo Shen, Hainv Gao

**Affiliations:** ^1^The First Hospital of Zhuji, 122nd Huansha Nan Road, Zhuji 311800, China; ^2^The State Key Laboratory of Diagnosis and Treatment for Infectious Disease, College of Medicine, The First Affiliated Hospital, Zhejiang University, 79th Qingchun Road, Hangzhou 310003, China

## Abstract

Dilutional hyponatremia is common in decompensated cirrhosis and can be successfully treated by tolvaptan, a vasopressin V2-receptor antagonist. Data were lacking regarding the effects of tolvaptan on cirrhotic patients with a Child-Pugh score of >10 and a serum sodium concentration of <120 mmol/L. We report a case of forties man with a 20-year history of chronic hepatitis B presenting with yellow urine and skin. Laboratory tests demonstrated prolonged prothrombin time, markedly elevated total bilirubin, severe hyponatremia, and a Child-Pugh score of >10. The patient was diagnosed with dilutional hyponatremia and was treated with recommended dosage tolvaptan at first. The serum concentration of sodium recover but the patient felt obviously thirsty. As the dosage of tolvaptan was decreased accordingly from 15 mg to 5 mg, the patient still maintained the ideal concentration of serum sodium. This case emphasizes that cirrhotic patient with higher Child-Pugh scores and serum sodium concentration of <120 mmol/L can be treated with lower dose of tolvaptan.

## 1. Introduction

Dilutional hyponatremia is defined as a serum sodium concentration of <135 mmol/L. This condition is common in decompensated cirrhosis, with an incidence as high as 49% [1]. Recent studies have shown that hyponatremia is not only a sign of disease severity but also a direct factor that could aggravate the disease. In addition, acute hyponatremia is considered as an independent predictor of mortality in patients with cirrhosis [2, 3]. However, traditional therapies such as fluid restriction and supplementation of high-dose sodium show little effect. A prospective randomized study showed that only 0−26% of patients treated with water restriction had serum sodium concentrations of >5 mmol/L [3]. Patients feel thirsty and have difficulty in finishing water restriction therapy too. The efficacy of high-dose sodium replacement is limited and the replacement can aggravate ascites and edema as well. Therefore, sodium administration is not recommended for treatment of dilutional hyponatremia in cirrhotic patients [4]. Recently, it was reported that tolvaptan, a vasopressin V2-receptor antagonist, had significant effect on the therapy of hyponatremia in cirrhotic patients. Nevertheless, few data are available regarding the effects of tolvaptan on cirrhotic patients with a Child-Pugh score of >10 and a serum sodium concentration of <120 mmol/L. Herein, we present a case of a decompensated cirrhotic patient with a serum sodium concentration of 117 mmol/L who received low-dose tolvaptan.

## 2. Case Presentation

A 47-year-old man with a 20-year history of chronic hepatitis B was hospitalized with the complaint of yellow urine and skin for 5 months. The patient had been treated with lamivudine in combination with adefovir for 1 year and discontinued treatment of his own 10 months ago. Five months ago, he developed the symptoms of yellow urine and skin and followed by being diagnosed as decompensated hepatitis cirrhosis B and treated with entecavir. During entecavir treatment, he had a total bilirubin concentration of 200−350 *μ*mol/L, prothrombin time of 20−31 s, and a Child-Pugh score of >10. The bilirubin level was persistently high despite repeat (up to four times) artificial liver support therapy. Meanwhile, during the course of treatment, the patient showed hepatic encephalopathy, a large amount of ascites, and hyponatremia with numerous serum sodium concentrations as low as 115 mmol/L. He received large dosage of diuretic, lower salt intake (less than 2 grams per day), water restriction to less than 500 mL per day, and one time paracentesis, but the symptom was not relieved. The patient was treated with tolvaptan at an initial dose of 15 mg qd. After therapy, he had a urine volume of 6100 mL and became obviously thirsty, although the serum sodium concentration showed significant recovery. The tolvaptan dosage was decreased to 7.5 mg qd, but he remained thirsty. For the large volume of urine and the obvious thirst, the dosage of diuretic was decreased once but resumed soon because of increasing ascites. Finally, the dosage of tolvaptan was maintained at 5 mg qd, and the patient's serum sodium concentration was consistently 128 mmol/L and daily urine volume was between 3000 and 4000 mL (see Table 1). During the course of therapy, the condition of patient was markedly improved, as indicated by the continuously decreased level of total bilirubin. After 1 month of treatment with tolvaptan, the drug was stopped and the serum sodium concentration was maintained at 130 mmol/L. Coused drugs were entecavir tablet, ademetionine injection, zolpidem tartrate tablet, lactulose, magnesium isoglycyrrhizinate injection, albumin injection, piperacillin-tazobactam injection, and ornithine aspartate injection during the therapy of tolvaptan. The patient was discharged from the hospital with a total bilirubin concentration as low as 94 *μ*mol/L and a serum sodium concentration of 135 mmol/L. From that time, the patient stopped diuretics therapy and just used entecavir. At the 3-month follow-up, total bilirubin concentration was only 38 *μ*mol/L and serum sodium concentration was 137 mmol/L. B ultrasound showed small amount of ascites. At the 1-year follow-up, the liver function of the patient was normal and B ultrasound showed no ascites at all.

## 3. Discussion 

We presented a middle-aged man with a history of 20-year chronic hepatitis B who presented with yellow urine and skin. Laboratory tests demonstrated prolonged prothrombin time, markedly elevated total bilirubin, severe hyponatremia, and a Child-Pugh score of >10. The patient was diagnosed with dilutional hyponatremia and was successfully treated with low-dose tolvaptan.

Dilutional hyponatremia is the most common complication of cirrhosis. The Study of Ascending Levels of Tolvaptan in Hyponatremia 1 and 2 (SALT-1 and SALT-2) trials demonstrated that when tolvaptan was used at the dosage between 15 and 60 mg/d for 30 days, serum sodium concentrations can be resumed in most patients. In addition, the most common adverse event was thirst, and rapid correction of the serum sodium concentration did not occur [5]. However, it should be noted that only 63 patients with cirrhosis were enrolled in these 2 trials and those with a Child-Pugh score of >10 or a serum sodium concentration of <120 mmol/L were excluded. Thus, more clinical experience is required to investigate the effects of tolvaptan on cirrhotic patients with Child-Pugh scores of >10.

Recently, several studies have evaluated the effects of tolvaptan at lower doses for the treatment of cirrhosis. In a double-blind, parallel-group, multicenter phase III clinical trial in Japan [6] which aimed to verify the efficacy of low-dose tolvaptan in patients with liver cirrhosis-associated ascites and insufficient response to conventional diuretic treatment and investigate its pharmacokinetic and pharmacodynamic profiles, a total of 40 patients with cirrhosis and an average concentration of initial serum sodium for all patients being >120 mmol/L were included. 20 patients belonged to Child-Pugh Class C. The results showed that tolvaptan at a dose of 7.5 mg/d could increase the urine output and decrease the ascitic volume. The serum sodium concentrations were increased significantly on the first day. In our case, low-dose of tolvaptan was successfully used to treat a patient with an initial serum sodium concentration as low as 117 mmol/L and a Child-Pugh score of >10. Tolvaptan showed good safety since the blood pressure of patient was in the normal range during the therapy.

There are several possible reasons for the successful treatment of hyponatremia in cirrhosis with low-dose of tolvaptan. Firstly, it is reported that 99% of tolvaptan molecules bind to plasma proteins after entry into the bloodstream. For patients with cirrhosis, the serum albumin concentration was low due to decreased protein synthesis, which could result in a reduced protein-tolvaptan binding rate and an increase in free tolvaptan plasma concentration. Moreover, albumin levels vary in healthy individuals by 10% [7], which could lead to altered drug efficacy. Secondly, tolvaptan is primarily metabolized by CYP3A4 and the activity of CYP3A4 enzymes is changed in cirrhotic patients [8]. Thirdly, portal-systemic shunting in patients with advanced cirrhosis could reduce the first-pass effect of drugs and lead to a significant increase in absorption. The above effects might account for the improved efficacy of low-dose tolvaptan in patients during decompensation of liver function. To date, there is no simple endogenous marker to predict hepatic function with respect to the elimination capacity of specific drugs and guide dose adjustment in patients with liver injury. The semiquantitative Child-Pugh score is frequently used to assess the severity of liver function impairment. However, the Child-Pugh score only offers rough guidance for dosage adjustment and more sensitive markers need to be developed to guide drug dosage adjustment in patients with hepatic dysfunction. Finally, we cannot ignore that the concentration of serum sodium of this patient (about 130 mmol/L) did not reach normal level during the tolvaptan therapy. Since hyponatremia develops slowly and cirrhotic patients show good hyponatremia tolerance, 130 mmol/L might be enough for the chronic dilutional hyponatremia patient.

The improvement of hyponatremia such as reduced occurrence of hepatic encephalopathy [9, 10] and improved quality of life [11] and prognosis of cirrhosis [12] may also lead to clinical benefits in patients. In our study, the hyponatremia correction was accompanied by gradual improvement of liver function and the Child-Pugh score. However, further studies are needed to investigate the clinical benefits of tolvaptan therapy after the correction of dilutional hyponatremia. In contrast, a recent meta-analysis indicated that the treatment of dilutional hyponatremia with vaptans did not result in a good prognosis. Twelve randomized, controlled trials with a total of 2,266 patients were included in this analysis, and the main outcome measures were mortality, spontaneous peritonitis, hepatic encephalopathy, and upper gastrointestinal hemorrhage. The results showed that vaptans could significantly increase serum sodium levels and lead to reduction in weight, whereas there was no clear difference between vaptans and placebo groups regarding prognosis [13]. During the therapy, we cannot ignore another phenomenon that although the urine volume was large, we still cannot decrease the dosage of diuretic or stop it during the therapy of tolvaptan. This may contribute to different mechanisms of drugs action.

The results of the present study suggested that, for cirrhotic patients with higher Child-Pugh scores (Class C) and serum sodium concentrations of <120 mmol/L, low-dose tolvaptan is effective for gradually increasing serum sodium concentrations, maintaining electrolyte balance, and possibly improving liver function. Further studies are needed to determine the optimal method of tolvaptan dose adjustment.

## Figures and Tables

**Table 1:** Serum sodium concentrations and urine volumes of the present patient under the treatment of different doses of tolvaptan.

Data (day)	Baseline∗	1	2	3	4	5	8	11	14	17	20	30	60	120	1 (yr)
Dosage of tolvaptan (mg)	NA	15	15	7.5	7.5	5	5	5	5	5	5		NA	NA	NA
Urine volume (mL)	2050	4400	6100	4050	4200	3100	2750	3000	3550	3800	2750	4300	4000	NA	NA
Serum sodium (mmol/L)	132	117	123	130	130	128	NA	128	NA	127	NA	129	135	NA	138
Total bilirubin (*μ*mol/L)	317	244	NA	246	NA	227	NA	196	NA	199	NA	157	92	38	35
ALT (U/L)	52	51	NA	NA	44	NA	NA	34	30	28	NA	33	17	20	17
PT (s)	27.3	23.2	NA	NA	25	NA	NA	24.1	24.9	23.8	NA	21.8	22.4	19.6	14.2
BP (mmHg)	124/69	114/64	NA	NA	NA	NA	107/62	104/72	114/68	NA	122/71	115/65	127/70	NA	125/65
Body weight (Kg)	76	67.5	65	66.5	66.5	69	NA	NA	65.5	NA	61	61.5	NA	NA	71
Child-Pugh score	14	14	NA	NA	NA	NA	NA	NA	NA	NA	NA	11	10	7	5

*Baseline: the data was from the first day when the patient was admitted.
